# Alcohol Use Among Adolescents

**Published:** 1998

**Authors:** Patrick M. O’Malley, Lloyd D. Johnston, Jerald G. Bachman

**Affiliations:** Patrick M. O’Malley, Ph.D., is a senior research scientist, Lloyd D. Johnston, Ph.D., is a distinguished research scientist, and Jerald G. Bachman, Ph.D., is a distinguished research scientist at the University of Michigan, Ann Arbor, Michigan

**Keywords:** AOD use behavior, adolescent, survey, prevalence, trend, demographic characteristics, minimum drinking age, attitude toward AOD, illicit drug, interpersonal AODR (alcohol and other drug related) problems, heavy AOD use, educational environment, causes of AODU (alcohol and other drug use), AOD associated consequences, high school student

## Abstract

Several ongoing national surveys, including the Monitoring the Future study, the National Household Survey on Drug Abuse, and the Youth Risk Behavior Survey, are investigating the drinking behaviors of adolescents in the United States. These studies have found that the majority of adolescents under the age of 18 have consumed alcohol, although the minimum legal drinking age is 21. Drinking rates may even have increased in recent years in some age groups. No substantial differences exist among various sociodemographic subgroups with respect to drinking rates, although alcohol consumption generally is lowest among African-Americans and highest among whites. Moreover, alcohol consumption increases sharply throughout adolescence. Various attitudinal and behavioral factors, such as religious involvement, truancy, and average grade level, also influence adolescents’ drinking behaviors. Almost two-thirds of 12th graders who report consuming alcohol experience at least one alcohol-related problem. Most adolescents drink to experience the pleasurable effects of alcohol, such as having a good time with friends.

Many American adolescents use alcohol, even though the minimum legal drinking age is 21. This article describes the extent and nature of alcohol use among American adolescents. In addition, the article provides information on trends in, self-reported reasons for, and consequences of adolescent alcohol use. The article draws on information from the Monitoring the Future (MTF) study, the National Household Survey on Drug Abuse (NHSDA), and the Youth Risk Behavior Survey (YRBS), which are described in the following section. The discussion emphasizes, however, findings from the MTF study (unless otherwise indicated, those findings are summarized in Johnston et al. 1998).

## Prevalence of Alcohol Use Among Adolescents

Several major ongoing national surveillance systems collect and evaluate information on alcohol use patterns among adolescents:

- The MTF study, which is conducted under a research grant to the University of Michigan from the National Institute on Drug Abuse
- The NHSDA, which is conducted by the Substance Abuse and Mental Health Services Administration (SAMHSA)
- The YRBS, which is conducted by the Centers for Disease Control and Prevention (CDC).

The MTF study, which uses a school-based sample, has conducted annual surveys of nationally representative samples of high school seniors since 1975 and of 8th and 10th grade students since 1991. It surveys about 50,000 students per year, using self-completed questionnaires administered under confidential conditions in classrooms. The study is designed to provide estimates of alcohol and other drug use and of related attitudes and beliefs among the Nation’s secondary school students.

The NHSDA surveys a nationally representative, household-based sample of Americans age 12 and older. The surveys are conducted as face-to-face interviews, with self-administered forms used for sensitive questions. In recent years, the surveys have been conducted annually. Youth samples have varied in size; the most recent survey, conducted in 1997, included 7,844 individuals ages 12 to 17. The NHSDA is designed to provide annual estimates of alcohol and other drug use among the Nation’s general population.

The school-based YRBS, which has been conducted biennially since 1991, surveys high school students in grades 9 through 12 using self-completed questionnaires administered under confidential conditions in classrooms. Sample sizes have varied; the 1997 survey included 16,262 students. The YRBS is designed to provide estimates of various risk behaviors, including some measures of alcohol and other drug use, among the Nation’s high school students.

Although none of the three surveillance systems is devoted entirely, or even primarily, to alcohol-related material, each includes data on alcohol use among American adolescents. The scope of this information, however, is somewhat limited. For example, although all three surveys evaluate patterns of recent alcohol use, information on other aspects of alcohol consumption (e.g., the respondents’ family histories of alcohol use and abuse, expectancies about alcohol use, methods of obtaining alcohol, and consequences of alcohol use) is sketchier.

Analyses of the most recent data from the three survey systems found high rates of alcohol use and relatively low rates of complete abstention among American adolescents, as follows:

- The 1997 MTF study found that 54 percent of 8th graders (almost all of whom are ages 13 to 14) reported having consumed alcohol (i.e., more than just a few sips) in their lifetime. The corresponding rates for 10th graders (i.e., ages 15 to 16) and 12th graders (i.e., ages 17 to 18) were 72 percent and 82 percent, respectively (Johnston et al. 1998).
- The 1996 NHSDA reported that 39 percent of adolescents ages 12 to 17 had drunk alcohol. The study also noted a sharp age-related increase in alcohol use, with 14 percent of 12- to 13-year-olds, 39 percent of 14- to 15-year-olds, and 62 percent of 17- to 18-year-olds reporting previous alcohol consumption (SAMHSA 1998*a*).
- According to the 1997 YRBS, 79 percent of students in grades 9 through 12 had consumed alcohol. In this survey, the rates of alcohol use increased steadily from 72 percent among 9th graders to 84 percent among 12th graders (CDC 1998).

Despite the consistent finding that relatively few Americans are complete abstainers by their late teens, substantial differences exist among the surveys with respect to the specific rates of alcohol consumption for the various age groups. Some of the differences may result from the specific design of each survey. For example, some studies include only students who are still in school (e.g., the MTF study), whereas other studies (e.g., the NHSDA) also include school dropouts, who may be at higher risk for alcohol use. Furthermore, the MTF study and the YRBS are school based, whereas the NHSDA is conducted in a home setting where parents may be present, which may lead to greater reluctance to report deviant behavior. Differences among the surveys in the wording of the questions and in the way in which the survey is administered (i.e., interview versus self-administered questionnaire) also may affect the outcome and account for some of the discrepancies in the findings obtained.

### Differences Among Demographic Subgroups

**Figure 1:**
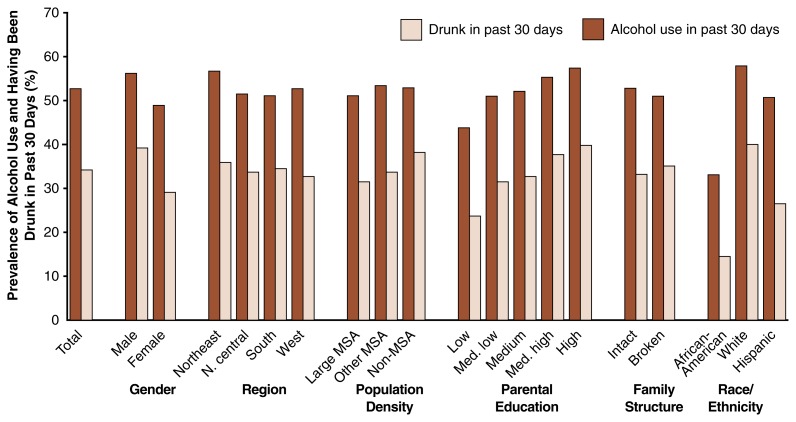
Percentage of 12th graders in various demographic subgroups who had used alcohol and had been drunk in the past 30 days. The students were surveyed in 1997 for the Monitoring the Future study. MSA = metropolitan statistical area. Intact family structure = students living with two parents. Broken family structure = students living with one or no parents.

The MTF study assesses both the prevalence of alcohol use and the prevalence of getting drunk among 8th, 10th, and 12th graders. To this end, respondents are asked, “Have you ever had any beer, wine, wine coolers, or liquor to drink—more than just a few sips?” Respondents who answer affirmatively then are asked: “On how many occasions have you had alcoholic beverages to drink—more than just a few sips . . . during the last 30 days?” and “On how many occasions (if any) have you been drunk or very high from drinking alcoholic beverages . . . during the last 30 days?” From these data, the prevalence of alcohol use and having been drunk can be determined for the entire sample as well as for various demographic subgroups based on gender, race, region of the country where the students live, population density of the area, parental education, and family structure. Among the most recent (i.e., 1997) results obtained for 12th graders, the following were particularly noteworthy (see table 1; also see figure 1 for demographic subgroup differences for 12th graders):

- The prevalence of alcohol use and of having been drunk were remarkably high. More than one-half of the 12th graders reported having had at least one drink—and more than one-third reported having been drunk at least once—in the past 30 days.
- Male students were more likely than female students to report drinking, although by only a relatively small margin (56 percent versus 49 percent). The gender difference was greater for having been drunk (39 percent versus 29 percent).
- The prevalence of both drinking and being drunk was highest in the Northeast region of the United States, but regional differences were rather small.
- Drinking rates differed only slightly by population density (i.e., metropolitan statistical area). Somewhat greater differences existed in the rates of being drunk, with students in more rural areas (i.e., counties where the largest city has a population less than 50,000) exhibiting the highest rates.
- Higher parental education (which serves as a proxy for higher socioeconomic status) was associated with increased rates of alcohol use and being drunk. Conversely, the association between family structure (operationalized as whether the student reported living with two parents versus one or no parents) and drinking status was weak and inconsistent.
- With respect to the three largest racial/ethnic subgroups, the rates of drinking and being drunk were lowest among African-American 12th graders, highest among white 12th graders, and intermediate among Hispanic 12th graders (see figure 1). Reliable national estimates of alcohol use among other racial/ ethnic subgroups (e.g., Asian-Americans or Native Americans) are more difficult to obtain, because their numbers are too low in national surveys. Bachman and colleagues (1991), using data from the 1976 to 1989 MTF surveys, showed that Asian-American 12th graders had low rates of alcohol use, whereas Native American 12th graders had relatively high rates of use. Similarly, combined data from the 1991 to 1993 NHSDAs have indicated that Asian-American youth ages 12 to 17 have lower rates of alcohol use (as measured by prevalence of use in the past year) than do African-American youth (SAMHSA 1998*b*). Even with the combined data, the NHSDA did not include enough Native American youth respondents to characterize that population.

Taken together, the data indicate that adolescent alcohol use has permeated all sociodemographic subgroups of society to a similar extent: In general, only minor differences exist among sociodemographic subgroups defined by geographical region, population density, parental education, and family structure. Those minor variations are less than they were 20 years ago, when differences were substantial, particularly with respect to region and population density.

### Developmental Differences

The prevalence of alcohol use and of having been drunk increases sharply during adolescence; rates are substantially higher among 12th graders than among 8th graders. Nevertheless, a considerable proportion of 8th graders already uses alcohol, with one-fourth of the students reporting having had a drink in the past 30 days. Moreover, one in three of those drinkers (i.e., one-twelfth of the total sample) reported having consumed enough alcohol to get drunk or very high.

Developmental differences also existed in the associations of various sociodemographic factors with drinking behavior. For example, in 8th grade, little difference existed between boys and girls in their rates of drinking (25 percent for boys versus 24 percent for girls) or having been drunk (8.4 percent for boys versus 7.9 percent for girls). By 12th grade, however, substantial gender differences in drinking behavior emerged. Similarly, parental education levels, which were positively related to alcohol use among 12th graders, were negatively related to alcohol use among 8th graders: Students with higher parental education were less likely to drink in 8th grade than were students with lower parental education. Among 12th grade students, however, drinking levels were higher among those with higher parental education. This change in the relationship between drinking level and parental education likely results at least in part from the fact that some students drop out of school before 12th grade. Those students are more likely to (a) use alcohol and get drunk and (b) come from homes with lower parental education levels than are students who stay in school. Consequently, if school dropouts were included in the 12th-grade surveys, the association of drinking levels with parental education would be less positive.

Among the three largest racial/ethnic groups evaluated in the MTF study, African-American students reported the lowest rates of drinking and getting drunk at all three grade levels. Hispanic and white students had similar rates in 8th grade, but a difference in the rates of getting drunk emerged by 10th grade, with whites having higher rates. This difference increased further by 12th grade. Analyses reported elsewhere support a “school dropout” effect as the most likely interpretation for the emerging difference (Wallace et al. 1995). This hypothesis posits that substantially higher dropout rates between 10th and 12th grade among Hispanic youth compared with white youth produce a more biased sample of Hispanic youth by 12th grade, because dropouts are more likely to use alcohol or get drunk (SAMHSA 1998*a*).

### Differences Based on Behavioral and Attitudinal Variables

**Figure 2:**
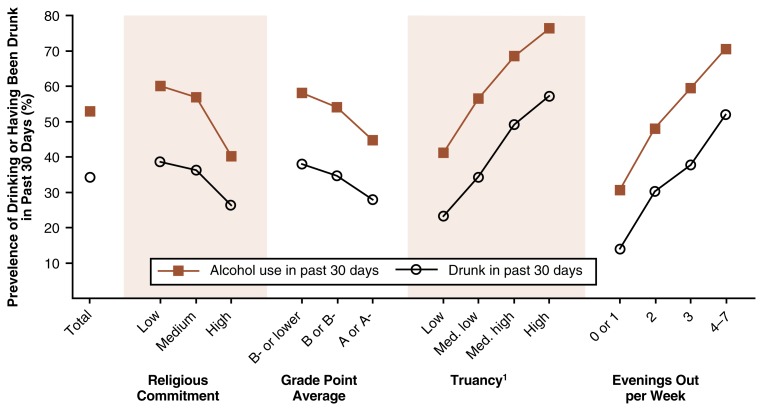
Percentage of 12th graders with various behavioral or attitudinal characteristics who had used alcohol or had been drunk in the past 30 days. The students were surveyed in 1997 for the Monitoring the Future study. ^1^For definiton of truancy, see table 2.

The MTF study also analyzed the prevalence of drinking or having been drunk in subgroups of adolescents based on several behavioral and attitudinal variables (i.e., religious commitment, grade-point average [GPA], truancy rates, and evenings out per week) (see table 2). Each of those variables was associated rather strongly with the adolescents’ drinking behaviors, either in a positive or a negative manner. Moreover, in contrast to the socio-demographic factors, the influences of these variables were similar for all three age groups, although the associations tended to get stronger with increasing age (see figure 2).

The behavioral factor that exhibited the strongest association with drinking behavior was the number of evenings that respondents reported going out for fun and recreation in a typical week. Thus, among 12th graders, 52 percent of those who went out 4 or more evenings a week reported getting drunk during the past 30 days, compared with 30 percent of those who went out 2 evenings a week and 14 percent of those who went out fewer than 2 nights a week. Similar differences already existed among 8th graders, with rates of getting drunk of 15 percent, 7 percent, and 4 percent among those who went out 4 or more nights, 2 nights, or fewer than 2 nights per week, respectively.

A similar positive association existed between truancy rates and alcohol use: Students with high truancy rates were far more likely than students with low truancy rates to be drinkers or to get drunk. For example, highly truant 12th graders were 2.5 times as likely as 12th graders with low truancy rates to report having been drunk in the past month (57 percent versus 23 percent).

In contrast to the frequency of going out and truancy, the students’ religious commitment (as determined by how important religion is to the student and how often he or she attends religious services) and GPA were negatively associated with the prevalence of drinking and being drunk. For example, only 40 percent of 12th graders with a high degree of religious commitment reported having drunk any alcohol in the past 30 days, compared with 60 percent of students with a low religious commitment. A similar relationship existed with respect to GPA: Whereas 45 percent of 12th grade students whose GPA was “A” drank alcohol in the past 30 days, 58 percent of students with a GPA of “B-minus” or lower did so. The association between GPA and drinking behavior was even stronger among 8th grade students, where approximately twice as many of the students with lower GPAs had consumed alcohol compared with those with the highest GPAs.

## Association Between Alcohol Use and Use of Other Drugs

One important reason for concern about adolescent alcohol use is its close association with the use of other drugs. There is considerable evidence that alcohol use tends to precede use of illicit drugs, and some researchers have argued, based on longitudinal data, that alcohol use serves as a “gateway” to the use of illicit substances (Kandel 1980; Kandel and Yamaguchi 1993). Analyses of the MTF study data have demonstrated that the cross-sectional association between alcohol and other drug use also is strong (see table 3). For example, among 8th graders who had not consumed alcohol at any time in their lives, only 3 percent had smoked cigarettes in the past 30 days or used marijuana in the past 12 months, and fewer than 0.5 percent had used cocaine in the past 12 months. Among 8th graders who had consumed alcohol at least 40 times in their lives, in contrast, approximately two-thirds had smoked cigarettes in the past 30 days or used marijuana in the past 12 months, and 18 percent had used cocaine in the past 12 months. Similar associations between alcohol consumption and other drug use existed among both 10th and 12th graders.

## Problems Caused by Adolescent Alcohol Use

Another reason for concern about adolescent alcohol use is the risk of serious social, medical, and legal problems that can result from alcohol consumption, such as impaired performance at school or work; interpersonal problems with friends, family members, teachers, and supervisors; physical and psychological impairment; and drunk driving. To assess the prevalence of such problems among alcohol-consuming adolescents, the MTF survey asked 12th graders whether alcohol had ever caused them any of 15 potential problems. Among the students in the 1994 to 1997 surveys who had consumed alcohol on at least 10 occasions during their lifetimes (i.e., 53 percent of all 12th graders surveyed), almost two-thirds (i.e., 62 percent) had experienced one or more of those problems (see table 4). Specifically, approximately 15 percent of the drinking 12th graders reported one problem, 13 percent reported two problems, and 34 percent reported three or more problems. Thus, a remarkable 18 percent of all 12th graders (i.e., 34 percent of 53 percent who had consumed alcohol on 10 or more occasions) had experienced 3 or more different alcohol-related problems, despite the fact that virtually none of them had reached the minimum legal drinking age. Similar results also were obtained in the NHSDA, which found that 38 percent of respondents ages 12 to 17 who reported drinking some alcohol in the past year had experienced at least one alcohol-related problem (SAMHSA 1998*a*).

The most common alcohol-related problem, which was reported by approximately one-half of the drinkers, was that alcohol use caused the respondent to behave in ways that he or she later regretted. Furthermore, almost one-third of the drinkers reported that alcohol use had interfered with their ability to think clearly. Another common and potentially serious problem was unsafe driving because of alcohol, which was reported by approximately one in five adolescent drinkers. Similarly common were alcohol-related interpersonal problems with significant others and parents.

## Reasons for Adolescent Alcohol Use

The MTF study also explored the reasons why so many young people drink alcohol. When asked for the most important reasons why they drank alcoholic beverages, 12th graders primarily emphasized the pleasurable aspects of drinking. For example, almost three-fourths of all 12th graders who had ever consumed alcohol gave “to have a good time with friends” as one of their reasons (table 5). Other commonly cited motivations related to alcohol’s pleasurable effects referred to alcohol’s good taste, its ability to make you feel good or high, and its ability to relax or relieve tension. Also high on the list of reasons for alcohol consumption was curiosity about alcohol and its effects, which was cited by approximately one-half of the respondents.

In contrast, substantially fewer adolescents reported using alcohol for coping with problematic situations. Thus, approximately one-fourth of 12th graders who ever consumed alcohol indicated that they drank because of boredom or because alcohol helped them escape their problems. This pattern of reasons for alcohol use is very similar to that given for marijuana use (Johnston and O’Malley 1986).

## Trends in Alcohol-Related Behaviors

**Figure 3:**
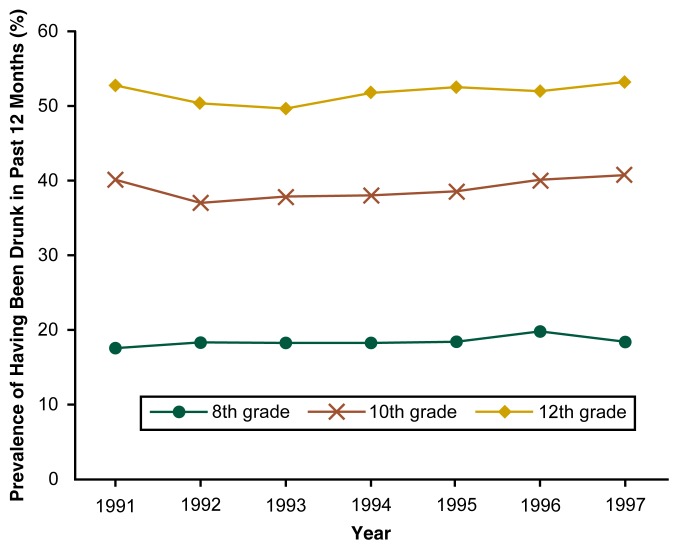
Trends in the percentage of 8th, 10th, and 12th graders who had been drunk during the past 12 months. The students were surveyed between 1991 and 1997 for the ongoing Monitoring the Future study.

**Figure 4:**
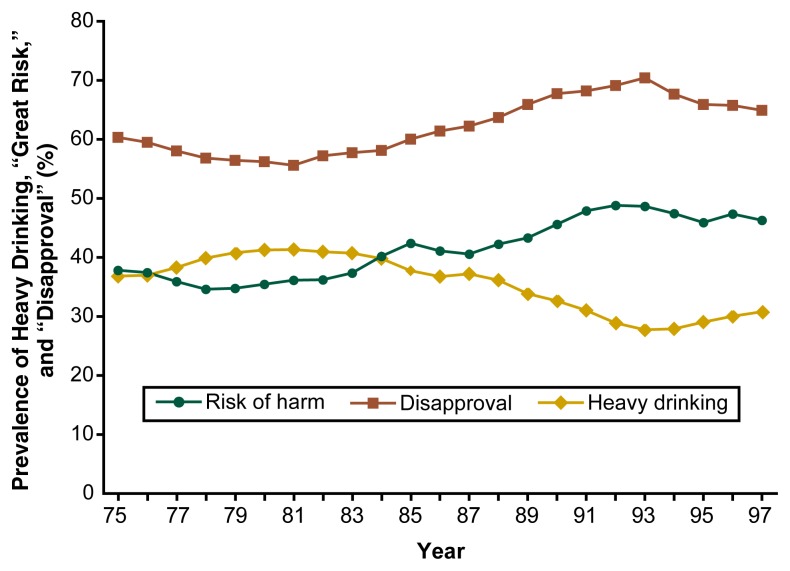
Trends in the percentage of 12th graders who had drunk heavily (i.e., five or more drinks on one occasion) in the past 2 weeks, who disapproved of heavy drinking, and who perceived a great risk of harm from heavy drinking. The students were surveyed between 1975 and 1997 for the ongoing Monitoring the Future study.

Recent trends in alcohol-related behaviors provide little cause for optimism regarding the current generation of American adolescents. For example, the percentages of 8th, 10th, and 12th graders who reported having been drunk at least once in the previous 12 months have not declined substantially within the past decade, but may, in fact, have increased in some age groups between 1992 and 1997 (figure 3). Similarly, the prevalence of another indicator of excessive adolescent drinking—heavy drinking (i.e., having five or more drinks in a row during the past 2 weeks)—appears to have increased among 12th graders in recent years, after declining consistently between 1982 and 1992 (figure 4). Conversely, two indicators of the beliefs and attitudes related to heavy drinking (i.e., disapproval of regular heavy drinking and the perception that heavy drinking is harmful) have declined since the early 1990s, after increasing during the 1980s. These findings suggest that a close association exists between adolescents’ attitudes toward and beliefs about drinking and their alcohol-related behavior.

## Conclusions

The findings presented in this article confirm that alcohol use and abuse, as well as alcohol-related problems, continue to be highly prevalent among American youth and a major source for concern. In fact, excessive alcohol use and its associated problems appear to have increased in recent years, following an earlier period during which both the rates of heavy drinking and the incidence of driving after drinking declined significantly among 12th graders. Moreover, the observations indicate that rates of alcohol use are equally high in almost all demographic subgroups. Finally, the beliefs and attitudes of adolescents toward drinking (and drinking and driving) show a close association with drinking behavior. What are the implications of these observations?

To date, researchers have not shown definitively whether changes in beliefs and attitudes actually play an active role in changing drinking behavior; whether changes in behavior produce changes in beliefs and attitudes about drinking; or whether other factors may affect beliefs, attitudes and behavior. Numerous analyses reported elsewhere, however, have supported the hypothesis that changes in attitudes and beliefs may have contributed to changes in the trends associated with both marijuana and cocaine use (for discussions of this hypothesis, see Bachman et al. 1998; Johnston et al. 1998). It is therefore very likely that attitudes and beliefs similarly play an important role in determining alcohol-related behaviors. If beliefs and attitudes indeed have important causal roles in shaping behavior, the next question becomes, How can society elicit changes in those beliefs and attitudes? Various aspects of the larger culture, including the mass media and advertising, influence the prevailing attitudes and behaviors, although the extent of their contributions is still controversial.

Alcohol-related public policies also help determine adolescent alcohol-use behaviors. For example, the increase in the minimum legal drinking age to 21 substantially reduced alcohol consumption as well as alcohol-related traffic fatalities among young Americans (O’Malley and Wagenaar 1991; Toomey et al. 1996). Other policies that have been suggested to modify adolescent drinking behaviors include “zero tolerance” laws, restrictions on the hours of permitted sales and the density of alcohol outlets (e.g., liquor stores and restaurants), and making retail licenses contingent on not selling to minors. Finally, some researchers have asserted that raising alcohol taxes can significantly reduce adolescent drinking (Chaloupka 1993; Grossman et al. 1994), although this hypothesis is still considered controversial.

The high levels of alcohol use and abuse, the recent disconcerting trends in these behaviors, and the erosion of antialcohol beliefs and attitudes among adolescents clearly indicate that more action is needed to address these problems on various fronts. Yet no consensus exists as to which specific actions should be undertaken. As more research results accumulate on the policies and programs aimed at deterring underage drinking, society’s responses to the problem of underage drinking should become increasingly effective.

## Figures and Tables

**Table 1:** Prevalence of Having Used Alcohol and of Having Been Drunk in the Past 30 Days Among Various Demographic Subgroups of 8th, 10th, and 12th Graders in 1997

	30-Day Prevalence of Alcohol Use (%)			30-Day Prevalence of Having Been Drunk (%)		
	
	Grade					

	8th	10th	12th	8th	10th	12th
	
**Total**	24.5	40.1	52.7	8.2	22.4	34.2
**Gender**						
Male	25.2	42.5	56.2	8.4	24.6	39.2
Female	23.9	37.9	48.9	7.9	20.2	29.1
**Region of Country**						
Northeast	24.8	41.1	56.7	7.9	21.9	35.9
North central	22.8	38.6	51.5	8.2	23.3	33.7
South	26.4	40.8	51.1	8.3	22.0	34.5
West	22.7	39.9	52.7	8.3	22.5	32.7
**Population Density**						
Large MSA*	23.1	37.8	51.1	6.6	20.7	31.5
Other MSA*	24.9	40.2	53.4	8.6	21.8	33.7
Non-MSA*	25.4	42.6	52.9	9.2	25.5	38.2
**Parental Education**						
Low	29.7	39.2	43.8	11.5	19.7	23.7
Medium low	26.2	41.1	51.0	9.3	22.5	31.5
Medium	27.8	41.6	52.1	10.2	24.1	32.7
Medium high	22.5	39.3	55.3	6.7	22.2	37.7
High	20.5	38.9	57.4	5.8	22.4	39.8
**Family Structure**						
Two parents	23.0	39.6	52.8	7.2	22.3	33.2
One or no parent	29.2	40.9	51.0	10.8	22.2	35.1
**Race/Ethnicity**						
African-American	16.9	26.2	33.1	3.1	9.9	14.5
White	25.7	42.8	57.9	9.0	25.8	40.0
Hispanic	30.1	40.0	50.7	9.8	18.6	26.5

* MSA = metropolitan statistical area.

SOURCE: Johnston et al. 1998.

**Table 2:** Prevalence of Having Used Alcohol and of Having Been Drunk in the Past 30 Days Among Various Behavioral and Attitudinal Subgroups of 8th, 10th, and 12th Graders in 1997

	30-Day Prevalence of Alcohol Use (%)			30-Day Prevalence of Having Been Drunk (%)		
	
	Grade					

	8th	10th	12th	8th	10th	12th
		
**Total**	24.5	40.1	52.7	8.2	22.4	34.2
**Religious Commitment**						
Low	32.3	48.1	60.0	13.0	30.7	38.5
Medium	30.0	45.3	56.8	10.3	25.9	36.2
High	18.5	30.3	40.2	5.0	14.5	26.3
**Grade Point Average**						
B- or lower	32.9	48.0	58.0	13.5	28.5	37.9
B or B+	23.9	38.3	54.0	7.5	20.7	34.6
A or A−	16.7	29.5	44.6	3.9	15.0	27.8
**Truancy**^1^						
Low	19.6	31.5	41.1	5.2	15.4	23.2
Medium low	42.4	52.4	56.4	15.8	30.7	34.4
Medium high	54.3	63.8	68.4	29.7	42.1	49.1
High	63.2	71.8	76.2	38.0	53.2	57.1
**Evenings Out per Week**						
0 or 1	15.6	25.0	30.6	3.7	11.1	14.0
2	23.5	37.9	48.1	6.6	19.4	30.1
3	29.3	46.6	59.4	10.4	25.0	37.6
4 or more	35.4	56.1	70.6	15.1	38.2	51.9

1 Levels of truancy are as follows: low = skipped 0 days and 0 classes in the past 4 weeks; medium low = skipped 1 day or 1 to 2 classes in the past 4 weeks; medium high = skipped 0 days and 3 to 10 classes, or 1 day and 1 to 5 classes, or 2 days and 0 to 2 classes, or 3 days and 0 classes in the past 4 weeks; and high = more than medium high.

SOURCE: Johnston et al. 1998.

**Table 3:** Prevalence of Cigarette, Marijuana, and Cocaine Use Among 8th, 10th, and 12th Graders With Various Levels of Lifetime Alcohol Use in 1997

	Lifetime Alcohol Use (Occasions)								

	8th Graders			10th Graders			12th Graders		
	None (*n*=8,066)*	1–39 (*n*=8,313)	40+ (*n*=1,083)	None (*n*=4,178)	1–39 (*n*=8,526)	40+ (*n*=2,225)	None (*n*=2,724)	1–39 (*n*=8,005)	40+ (*n*=4,196)
		
Used cigarettes in the past 30 days (%)	3	28	64	4	32	67	5	32	66
Used marijuana in the past 12 months (%)	3	26	66	5	39	76	4	33	72
Used cocaine in the past 12 months (%)	**	4	18	**	3	18	**	2	15

* Numbers of students are approximations.

** < 0.5 percent.

NOTE: An example for interpreting entries is as follows: Among the 8th graders who had used alcohol on 40 or more occasions in their lifetime, 64 percent had used cigarettes in the past 30 days.

SOURCE: Johnston et al. 1998.

**Table 4:** Alcohol-Related Problems Reported by 12th Graders Surveyed Between 1994 and 1997 Who Had Consumed Alcoholic Beverages on at Least 10 Occasions in Their Lifetime*

Alcohol-Related Problem Reported	12th Graders (%)
Caused you to behave in ways you later regretted	52
Interfered with your ability to think clearly	30
Caused you to drive unsafely	19
Hurt your relationship with your spouse, fiancé, or girlfriend/boyfriend	19
Hurt your relationship with your parents	16
Involved you with people you think are a bad influence	14
Caused you to have less energy	13
Hurt your relationships with your friends	12
Caused you to be less stable emotionally	11
Got you in trouble with the police	9
Hurt your performance in school and/or on the job	9
Caused you to be less interested in other activities than you were before	9
Caused your physical health to be bad	6
Had other bad psychological effect	4
Hurt your relationships with your teachers or supervisors	4

* Weighted number of cases = 4,952.

SOURCE: Johnston et al. 1998.

**Table 5:** Reasons for Alcohol Use Given by 12th Graders Surveyed Between 1994 and 1997 Who Had Ever Consumed Alcohol*

Most Important Reasons for Drinking Alcohol	12th Graders (%)
To have a good time with friends	73
To experiment, see what it’s like	52
To feel good, get high	46
Because it tastes good	46
To relax or relieve tension	44
Because of boredom, nothing else to do	25
To get away from my problems or troubles	23
Because of anger or frustration	17
To increase the effect of other drugs	8
To fit in with a group I like	8
To get to sleep	7
To seek insights and understanding	6
To get through the day	3
Because I am “hooked”	2
To decrease the effects of other drugs	1

* Weighted number of cases = 7,052.

SOURCE: Johnson et al. 1998.
